# Supplementary material for: Selection-driven trait loss in independently evolved cavefish populations
Source: Nat Commun. 2023 May 3;14:2557. doi: 10.1038/s41467-023-37909-8 (PMC10156726; doi:10.1038/s41467-023-37909-8)
Supplement: Supplementary file 1 — Supplementary Information [file 41467_2023_37909_MOESM1_ESM.pdf]

Supplementary Information for

Selection-driven trait loss in independently evolved cavefish populations

Author List

Rachel L. Moran*[1,2], Emilie J. Richards[1], Claudia Patricia Ornelas-García[3], Joshua B. Gross[4], Alexandra Donny[1], Jonathan Wiese[1], Alex C. Keene[5], Johanna E. Kowalko[6], Nicolas Rohner[7,8], and Suzanne E. McGaugh[1]

Affiliations

1. Department of Ecology, Evolution, and Behavior, University of Minnesota, Saint Paul, MN
2. Department of Biology, Texas A&M University, College Station, TX
3. Colección Nacional de Peces, Departamento de Zoología, Instituto de Biología, Universidad Nacional Autónoma de México, Tercer Circuito Exterior S/N. CP 04510, México, D. F. México.
4. Department of Biological Sciences, University of Cincinnati, Cincinnati, OH, USA 45221.
5. Department of Biology, Texas A&M University, College Station, TX
6. Department of Biological Sciences, Lehigh University, Bethlehem, PA
7. Stowers Institute for Medical Research, Kansas City, MO
8. Department of Molecular & Integrative Physiology, KU Medical Center, Kansas City, KS

*Corresponding author; email: rlmoran@tamu.edu

Supplementary Note 1

Population Structure

ADMIXTURE analysis revealed fine-scale patterns of genetic differentiation among populations. Of note, Japones and Yerbaniz cave populations grouped together, separate from the majority of the other El Abra cave populations (i.e., Jos, Montecillos, Palma Seca, Sabinos, Tigre, and Tinaja) which mirrors their geography, as they are less than 2 km from each other, possibly interconnected (Mitchell et al. 1977, Elliott 2018), and documented to share a genetic basis of the brown mutation (Gross et al. 2009). Pachón was also categorized as a distinct genetic group from the other El Abra caves. Chica and Toro, the two southernmost El Abra caves that are geographically very close to one another, were also grouped in this analysis. Admixed ancestry was evident in Toro, in agreement with previous work suggesting this cave contains recent hybrids between cave and surface fish (Panaram and Borowsky 2005). Admixed ancestry was not evident for the Chica cave population at K=11, but for some of the lower values of K (e.g., K=7) Chica individuals showed admixed ancestry with Río Choy surface (i.e., a proxy for Lineage 1 surface fish) and El Abra cave parental populations. This agrees with recent detailed analyses into the history of admixture in Chica cave (Moran et al. 2022). ADMIXTURE was also not evident in Subterráneo despite other approaches supporting admixture in this population (see below for further details). This discrepancy may be due to pronounced asymmetry in the relative genomic contributions from either parental population to the hybrid genomes (see below), as ADMIXURE has been shown to fail to identify hybrids under these circumstances (Kong & Kubatko 2021).

PCA on SNPs revealed genetic clustering of samples into eight clades, most of which contained only surface or only cave populations (Supplementary Fig. 4). PC1 accounted for 20.5% of the total variance and separated Lineage 1 from Lineage 2 populations. PC2 accounted for 13.2% of the total variance and separated the cave from the surface populations. Subterráneo cave was the only population that did not group with like ecotypes, instead clustering with several surface populations, including samples from Micos river. A tributary of the Micos surface population, Arroyo La Pagua, floods into Subterráneo cave during high water in the wet season, and it is common to observe surface fish in this cave (Elliott 2018). Thus, gene flow into Subterráneo cave from the surface could explain this pattern.

Notably, the Arroyo cave samples were split across two clusters, with some individuals grouping with old lineage cave populations, as expected from the geographic location of this cave, and other individuals clustering with Lineage 1 surface populations. This variation within samples from Arroyo is likely due to ongoing hybridization between this cave population and the local surface population. When fish were collected from Arroyo cave in 2019, putative hybrids were observed with some asymmetry in eye loss

and variation in body pigmentation and eye degeneration (P.O.-G., pers. obs.). Some surface fish, including cichlids, were also encountered in the cave pools, indicating a connection with the local surface waters, and some *Astyanax* cavefish showed parasites clearly translocated from the surface fish (P.O.-G., pers. obs.).

As expected given their smaller populations sizes and historical bottleneck events, cavefish populations tend to have much lower nucleotide diversity (pi) compared to surface fish populations (Supplementary Data 9, Supplementary Fig. 17). The genome-wide average for pi varied by an order of magnitude among all *A. mexicanus* populations, ranging from 0.00041 in Escondido cave to 0.0039 in the Mante surface population (Supplementary Data 9, Supplementary Fig. 17). Mean D_{XY} ranged from 0.00045 between Jineo and Molino caves (both Lineage 1 and in the Guatemala region) to an order of magnitude larger (0.0052) between Mante (Lineage 1) and Peroles (Lineage 2) surface populations (Supplementary Data 9, Supplementary Fig. 18a). These patterns are consistent with hypotheses surrounding the time since divergence and degree of gene flow between populations. Pairwise F_{ST} values (Supplementary Data 9, Supplementary Fig. 18b) largely reflect the high degree of variation in pi among populations rather than variation divergence, and thus, pairwise F_{ST} is not a reliable metric to quantify the divergence between populations in this system (Charlesworth 1998, Herman et al. 2018).

Supplementary Note 2

Cueva del Río Subterráneo Admixture Analyses

While our phylogenetic analyses suggested that Subterráneo cave may represent a third independent origin of cave adaptation in *Astyanax* (Fig. 1c, Supplementary Figs. 1-3), we found strong support for an alternative hypothesis. Specifically, our analyses indicated that recent admixture between the Subterráneo cave and the Micos surface population, a tributary of which (i.e., Arroyo La Pagua), floods into Subterráneo cave during high water in the wet season, has caused Subterráneo cavefish to group phylogenetically with surface fish populations rather than with other cavefish populations.

We inferred historical and contemporary migration events between Subterráneo cavefish, two Lineage 2 cave populations in the El Abra region (Pachón and Tinaja, representing caves at the northern and southern extent, respectively, of the El Abra cave region), a Lineage 2 surface population (Rascón), a Lineage 1 surface population (Mante), and an outgroup (*Astyanax nicaraguensis*). This analysis indicated historic gene flow events between Subterráneo and the El Abra caves (Pachón and Tinaja) and recent gene flow between Subterráneo and Mante, the Lineage 1 surface fish.

Formal tests for introgression using D and f4 statistics found significant support for introgression between all populations examined. For a given population trio, we observed that Subterráneo shares a higher percentage of derived alleles with the Lineage 1 cavefish population (Escondido: 33%) compared to the Lineage 2 cavefish populations (Pachón: 26%; Tinaja: 23%) (Supplementary Table 1). This could be interpreted to suggest that Subterráneo cavefish originated from Lineage 1 surface stock. However, ongoing hybridization with the local Lineage 1 Micos surface fish may artificially inflate the number of shared derived alleles present between Subterráneo cavefish and the Lineage 1 cavefish.

Fine-scale ancestry mapping using a HMM also indicated that Subterráneo individuals appear to have a highly admixed genome with ancestry from the Lineage 1 Micos surface population and Lineage 2/El Abra region caves (Supplementary Fig. 6), indicating that Subterráneo cave was originally populated by the same Lineage 2 surface stock that populated the El Abra caves. Notably, our analyses showed that the Subterráneo hybrid population is unique from other previously described hybrid populations in that the majority of ancestry is derived from surface fish (Mean ± SE percent surface ancestry = 84 ± 0.44). In contrast, hybrid populations in Chica and Caballo Moro caves were recently shown to have a majority of their genomes derived from cave ancestry, with only 15-25% of their genomes derived from surface fish ancestry (Moran et al. 2022, Medley et al. in review).

Supplementary Note 3

Inferring Recent Demographic History

Population genomic approaches used to detect signatures of selection commonly rely on demographic parameters inferred from the site frequency spectrum (SFS). We used a non-parametric method implemented in Stairway Plot 2 (Liu and Fu 2020) to estimate past changes in population size that may influence our ability to detect and classify regions of the genome that have experienced selective sweeps. We used previously generated unfolded SFS for Pachón cave, Tinaja cave, Molino cave, Río Choy, and Rascón populations (Herman et al. 2018). The white long fin tetra was used to infer the ancestral allele and polarize the data, which consisted of 500Mb of sequence (including invariant sites) for each population. The SFS for each population was provided to Stairway Plot 2 and singletons were masked. We used a generation time of 1 year and a mutation rate of 3.5e-9 estimated from cichlids (Malinsky et al. 2018).

Recently, (Herman et al. 2018) conducted demographic modeling in ∂a∂i (Gutenkunst et al. 2010) using 2D unfolded SFS to estimate present day effective population sizes and the timing of cave-cave, cave-surface and surface-surface (Lineage 1 and Lineage 2) population splits and timing of secondary contact

(and admixture) between populations. The present analysis used the 1D unfolded SFS for each population to provide estimates of population size changes over time, as well as bottleneck and expansion events in the recent past. Cave populations were expected to have experienced marked bottlenecks corresponding to the time that the surface population invaded caves. Previous demographic modeling with ∂a∂i indicated that surface and cave populations from both Lineage 1 and Lineage 2 split from one another at remarkably similar times (~160,000 generations ago for both lineages), and that the split between the two surface lineages occurred around 260,000 generations ago. Previous analyses also supported a model of secondary contact with ongoing gene flow between cave and surface populations (Herman et al. 2018). We note that Stairway Plot 2 does not account for ongoing gene flow between populations, so estimates of effective population sizes may be inflated compared to previously published estimates from ∂a∂i (Herman et al. 2018).

Our estimates of past population size changes with Stairway Plot 2 indicated consistent differences between cave populations (i.e., Pachón, Tinaja, Molino) and surface populations (i.e., Rascón and Río Choy) (Fig. 2a, Supplementary Fig. 12). Most notably, cave populations underwent a bottleneck corresponding to previously estimated timing of cave-surface population splits (i.e., invasion of caves by surface ancestors) between 100-200k generations ago (also supported by ∂a∂i analyses from (Herman et al. 2018)). Our stairway Plot 2 analysis revealed that both Lineage 2 (Rascón) and Lineage 1 (Río Choy) surface populations appear to have experienced a more ancient bottleneck event at approximately 800k generations ago, potentially corresponding to their migration into Northern Mexico, followed by subsequent population expansions.

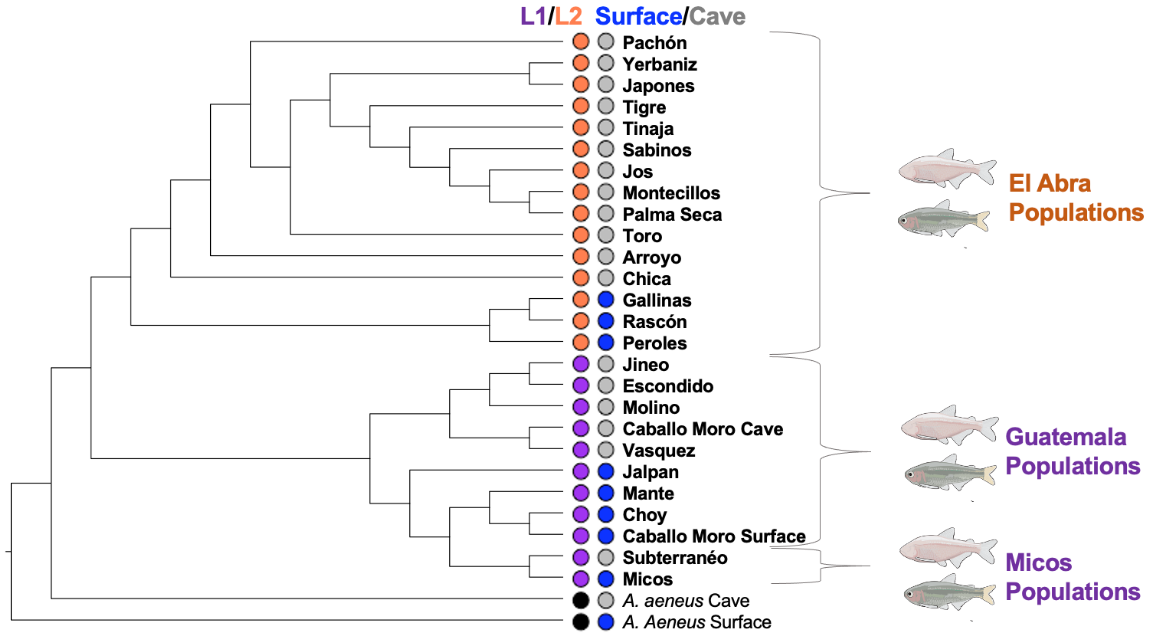

Supplementary Figure 1. **Phylogenomic signature of repeated evolution of cave adaptation in A. mexicanus.** Maximum likelihood population tree built in Treemix using 680,021 SNPs shared across all populations sampled.

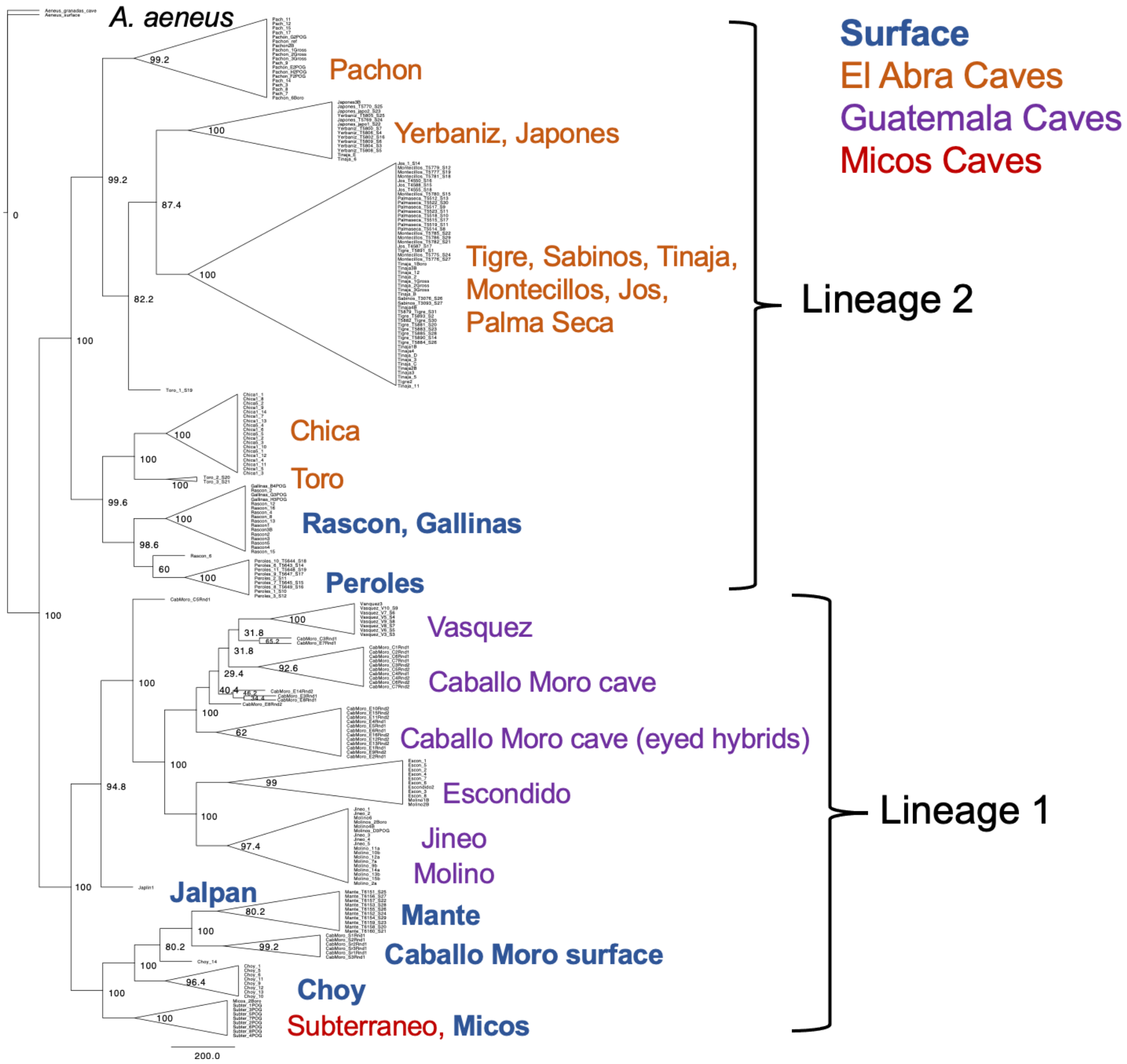

Supplementary Figure 2. Full multi-species-coalescent tree inferred in SVDQuartets with all samples shown. A total of 1,121,282 SNPs were used to infer the multi-species-coalescent tree in SVDQuartets. SVDquartets was run with a sampling of 500,000 random quartets and 500 standard bootstrap replicates specified to obtain bootstrap node support values.

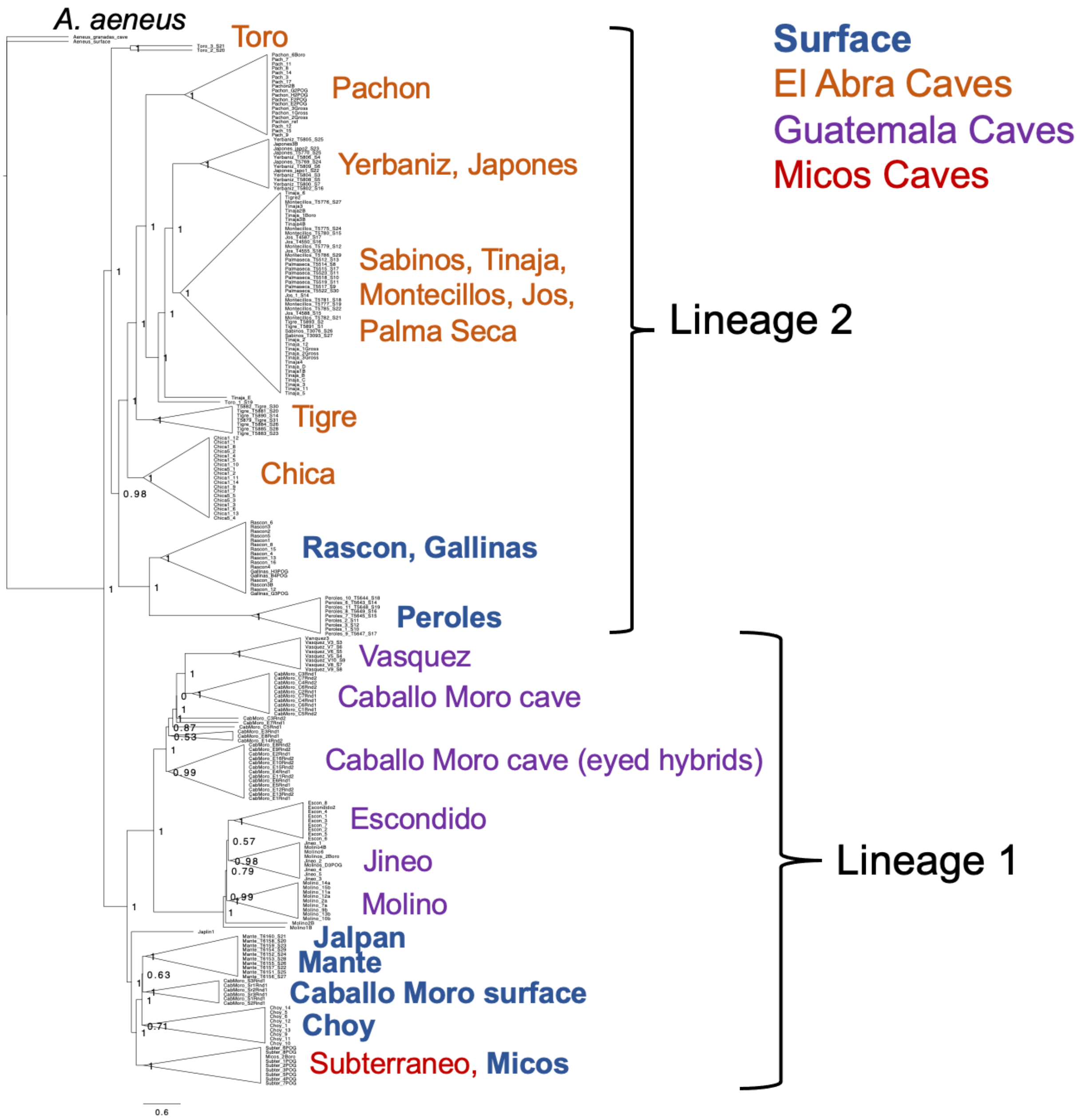

Supplementary Figure 3. Species tree inferred using a coalescent gene tree-based approach in ASTRAL. A total of 3,339 single copy orthologs covering 63,395,097 BPs total were used to infer this species tree in ASTRAL. Branch lengths are given in coalescent units and branch supports (node labels) are measured as local posterior probabilities. Final quartet score = 440574139629. Final normalized quartet score = 1.047.

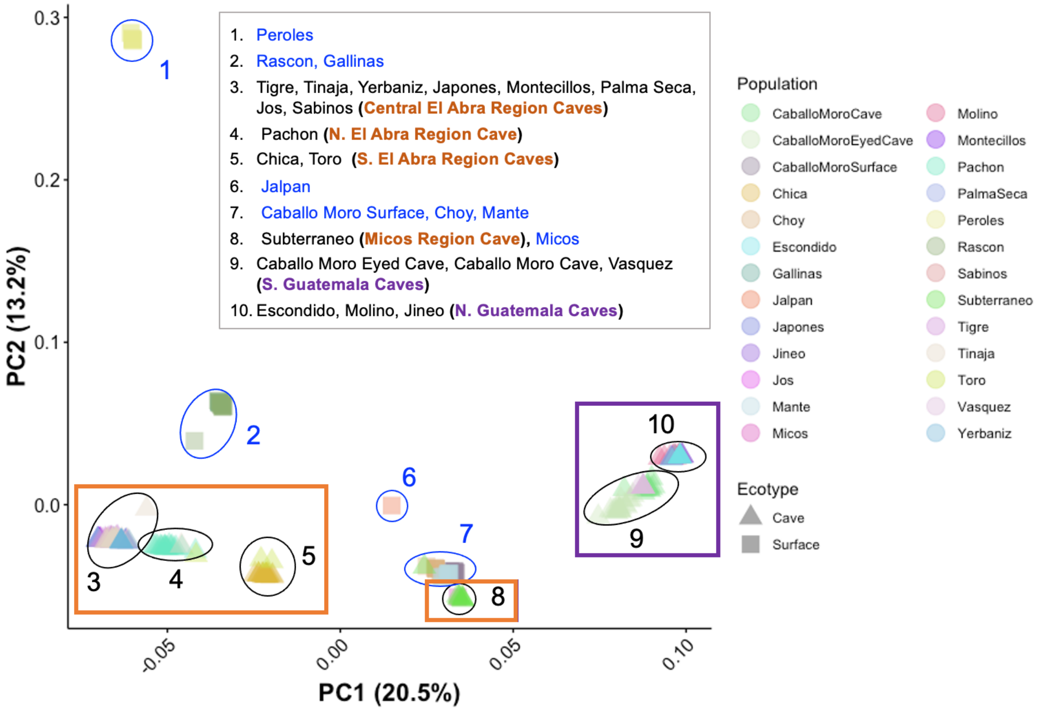

Supplementary Figure 4. Principal Component (PC) analysis on SNPs. This analysis used 751,759 SNPs across eight surface and 18 cave populations of *Astyanax mexicanus* (see Supplementary Data 1 for sample sizes). Each population is assigned a unique color (see legend). For surface populations, the collection location is provided in blue text in the inset and individuals are represented with squares. For cave populations, the collection location is provided in black text in the inset and individuals are represented with triangles. Additionally, the Micos and Guatemala caves are shown within purple boxes (these two cave regions contain cavefish originating from the same lineage of surface stock). The El Abra caves are shown within an orange box (this cave region contains cavefish from a second independent lineage of surface stock). Clustered populations are grouped in clades numbered 1-10 (indicated with ellipses; also see inset) based on overlapping PC values.

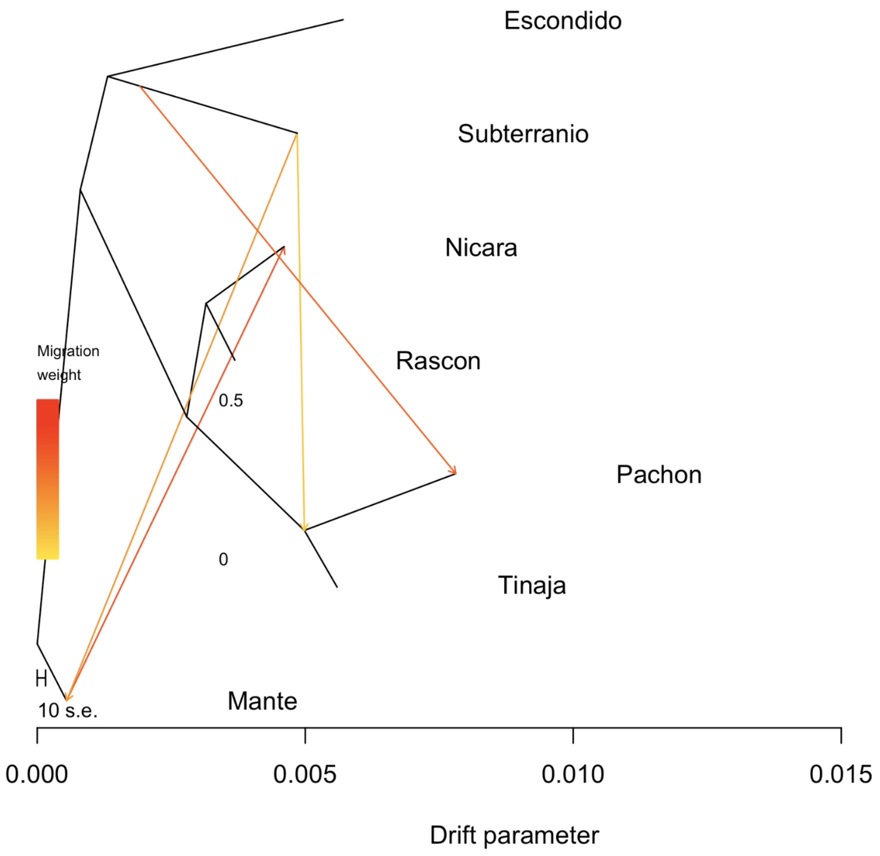

Supplementary Figure 5. Treemix graph showing phylogeny of select representative cave and surface populations and historical gene flow events. This graph depicts the phylogenetic relationship and inferred migration events (m=4) between Subterráneo, two Lineage 2 cave populations in the El Abra region (Pachón and Tinaja, representing caves at the northern and southern extent, respectively, of the El Abra cave region), a Lineage 2 surface population (Rascón), a Lineage 1 surface population (Mante), and an outgroup (Nicara = *Astyanax nicaraguensis*, surface outgroup congener from Nicaragua). Historic gene flow events are indicated between the El Abra caves (Pachón and Tinaja) with Subterráneo and recent gene flow is indicated between Subterráneo and Mante (representing Lineage 1 surface fish). Recent hybrids, as indicated by ADMIXTURE analysis, were removed from the data set prior to running additional analyses to detect introgression.

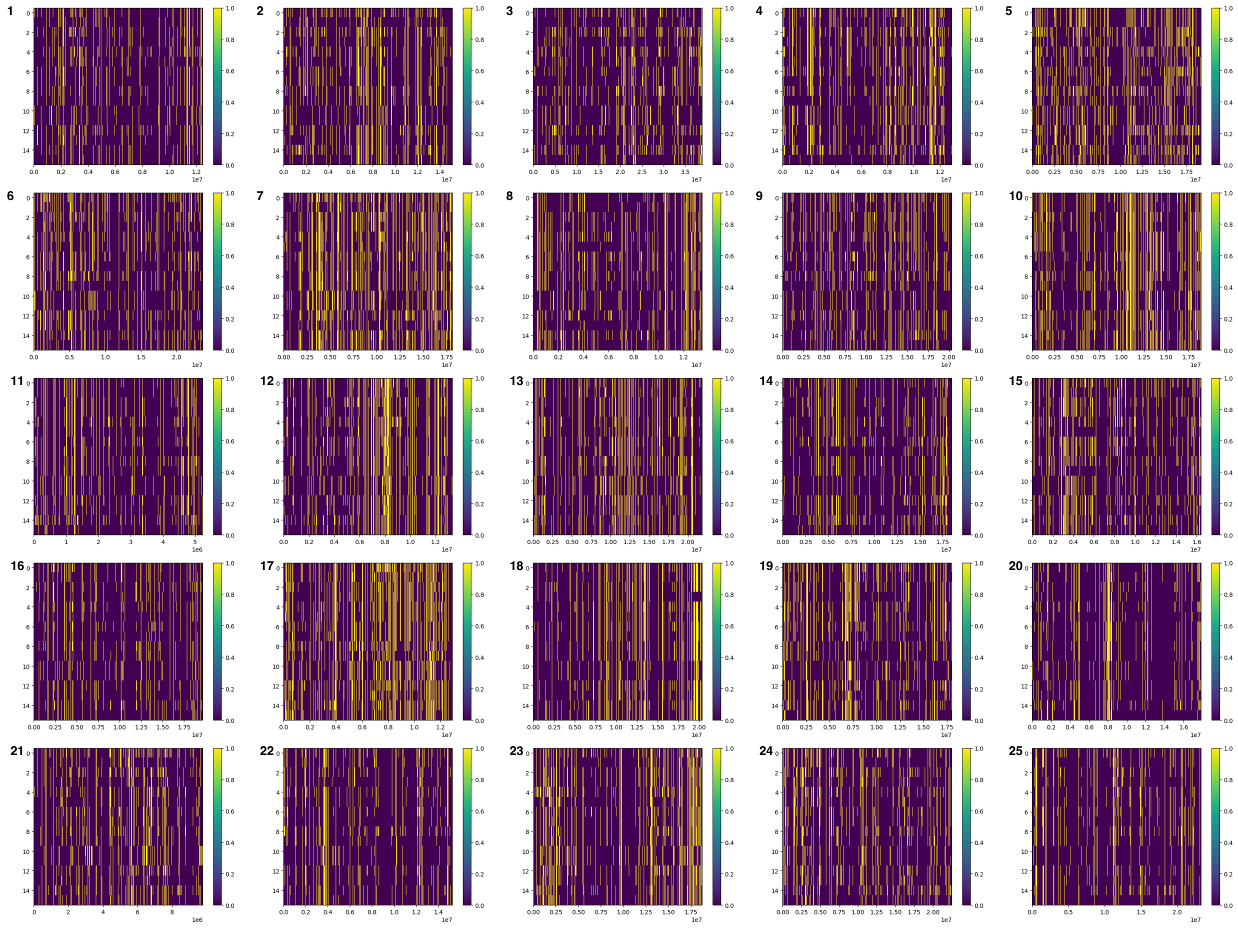

Supplementary Figure 6. Visualization of local ancestry tracts in Subterráneo samples. Local ancestry was inferred using a Hidden Markov Model approach along each of the 25 chromosomes. Pachón and Mante were used as the cave and surface parental populations, respectively. Yellow represents cave ancestry and purple represents surface ancestry. The y axis shows haplotypes 0-15 corresponding to n = 8 diploid individuals. The x axis shows bp position along each chromosome.

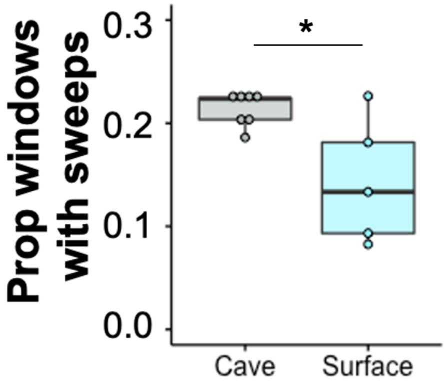

Supplementary Figure 7. Proportion of the genome under selection in cave and surface populations. A significantly higher proportion of 5 kb genomic windows were predicted by diploS/HIC to contain a hard or soft selective sweep in cave (n=7) compared to surface (n=5) populations. * indicates $p < 0.05$. Mean ± SE proportion of 5 kb windows under selection in surface populations = 0.143 ± 0.027; mean ± SE proportion of 5 kb windows under selection in cave populations = 0.213 ± 0.006; one-sided Wilcoxon rank sum test: W = 29, p-value = 0.037. For each box plot the horizonal line shows the median variant age, the shaded box spans the 25th to 75th percentile range, and the whiskers span the lowest to highest values that fall within 1.5 * the inter-quartile range. Raw data are shown over the box plots, with each dot representing a single population (cave: n = 7 populations; surface: n = 5 populations).

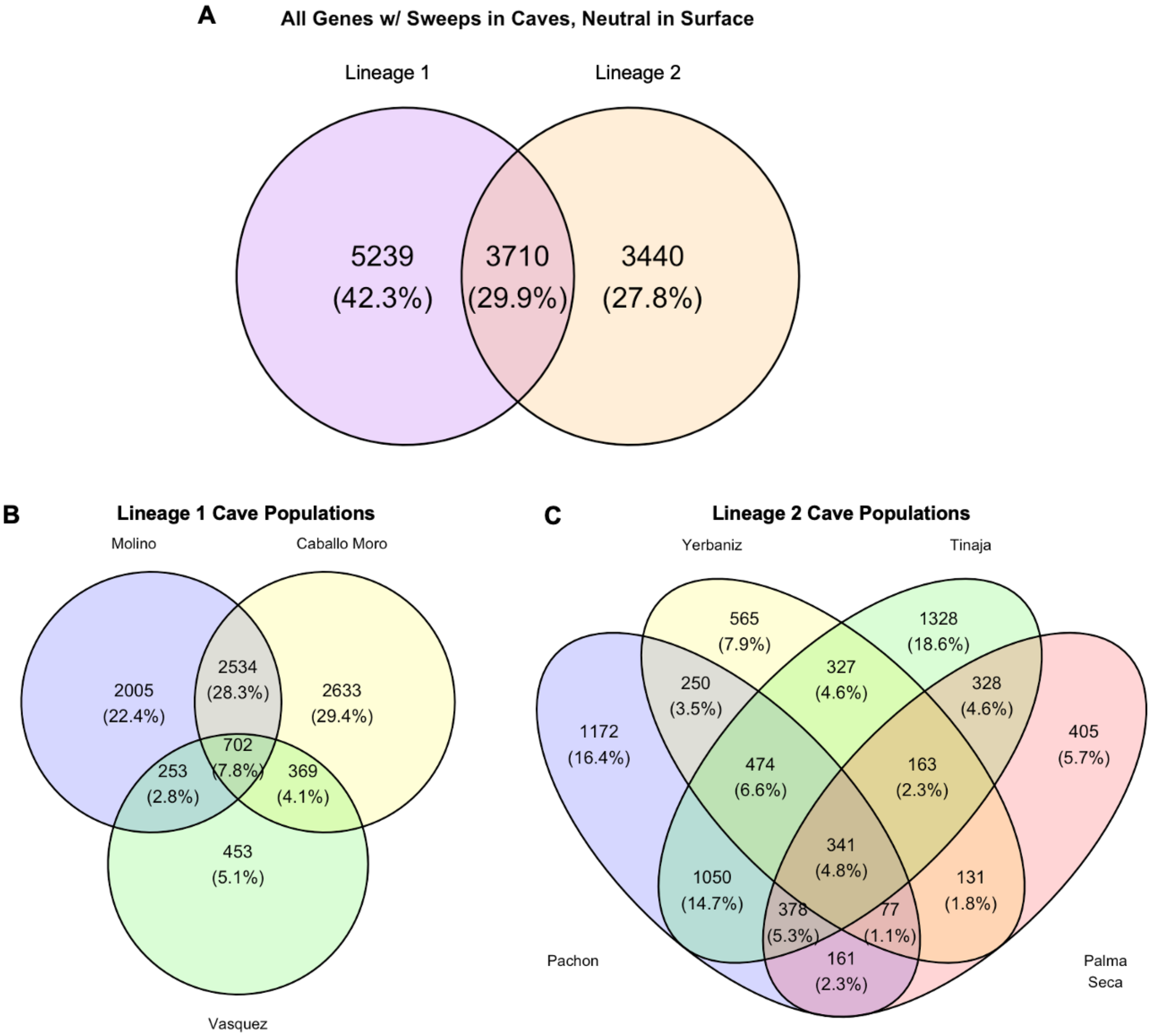

Supplementary Figure 8. Venn diagrams showing overlap in number of genes with a sweep in a cave population and neutral evolution in a same-lineage surface population. (A) All Lineage 1 and Lineage 2 cave populations are grouped together. (B) Comparison among Lineage 1 cave populations. (C) Comparison among Lineage 2 cave populations.

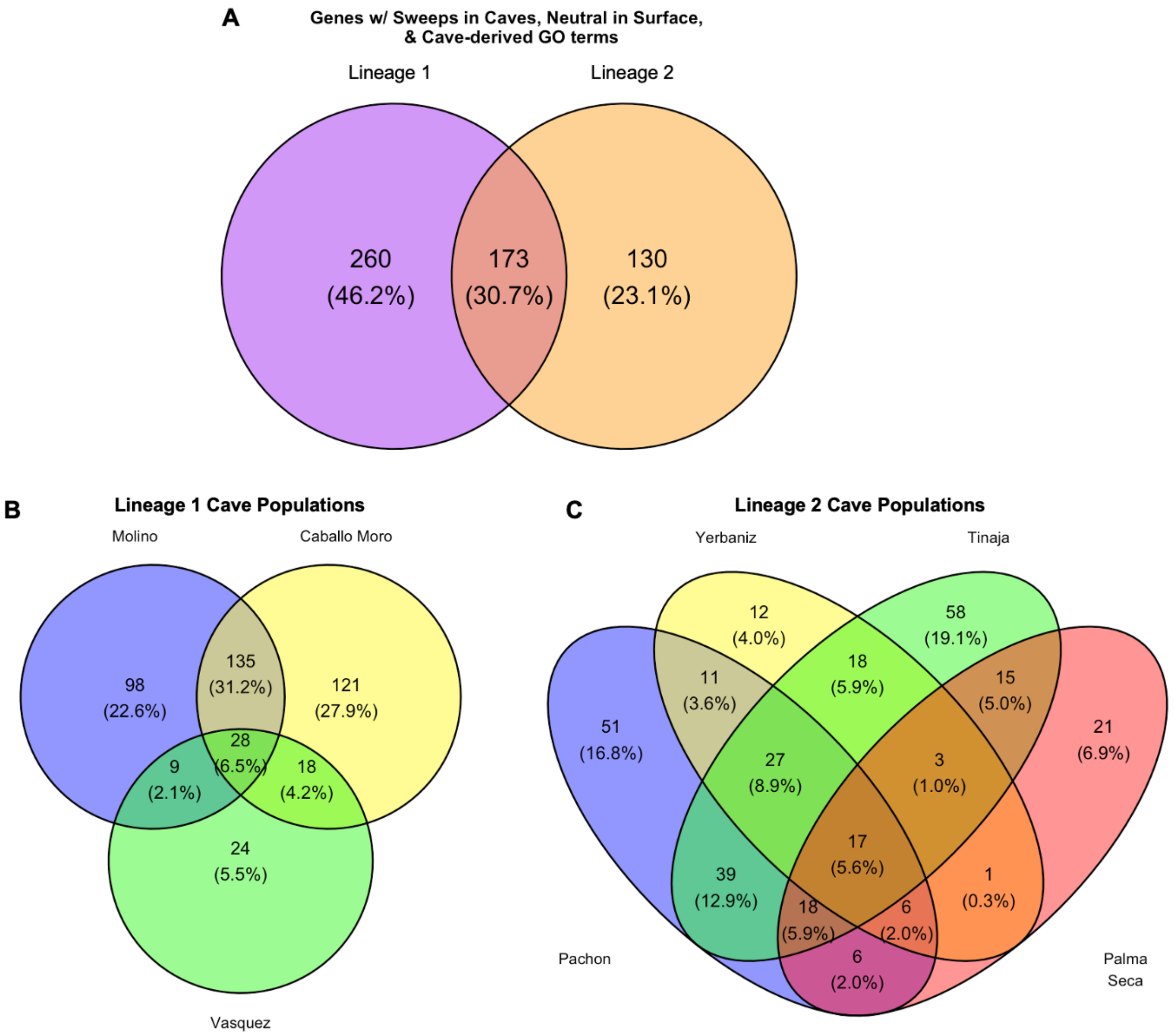

Supplementary Figure 9. Venn diagrams of cave-adaptive genes with GO-terms associated with cave-derived traits. (A) All Lineage 1 and Lineage 2 cave populations are grouped together. (B) Comparison among Lineage 1 cave populations. (C) Comparison among Lineage 2 cave populations.

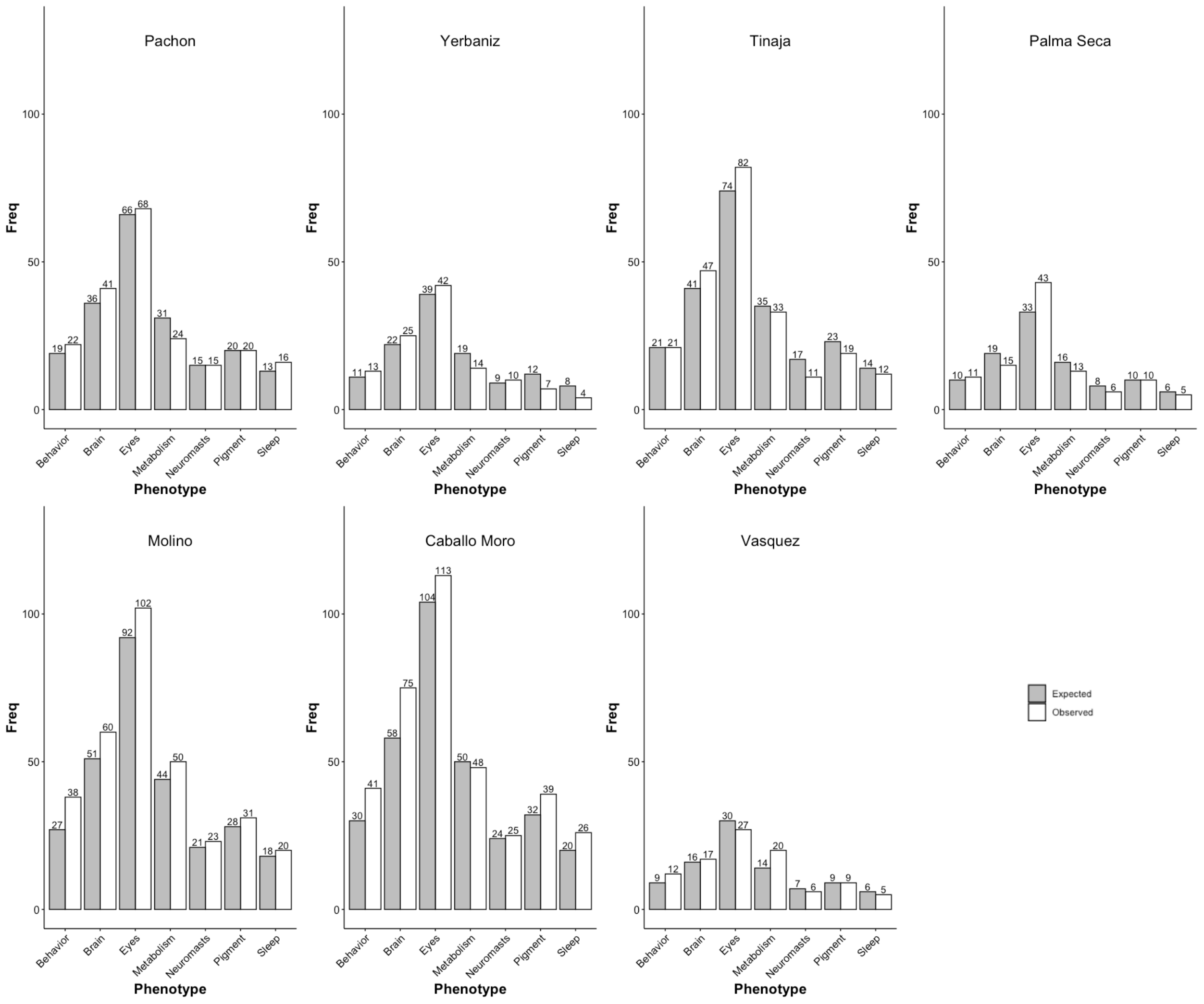

Supplementary Figure 10. Expected and observed genes with GO terms matching a given cave-derived phenotypic category. For each cave population, expected numbers for phenotypic categories were obtained by calculating the proportion of genes with GO terms in each phenotypic category in the entire surface fish genome annotation (26,698 gene total) and then multiplying by the total number of sweeps in each population. Fisher's exact tests were performed for each phenotypic category within each population and no significant differences were found between the expected and observed counts. Note that pleiotropic genes (i.e., those with GO terms associated with 2 or more phenotypic categories) were included multiple times.

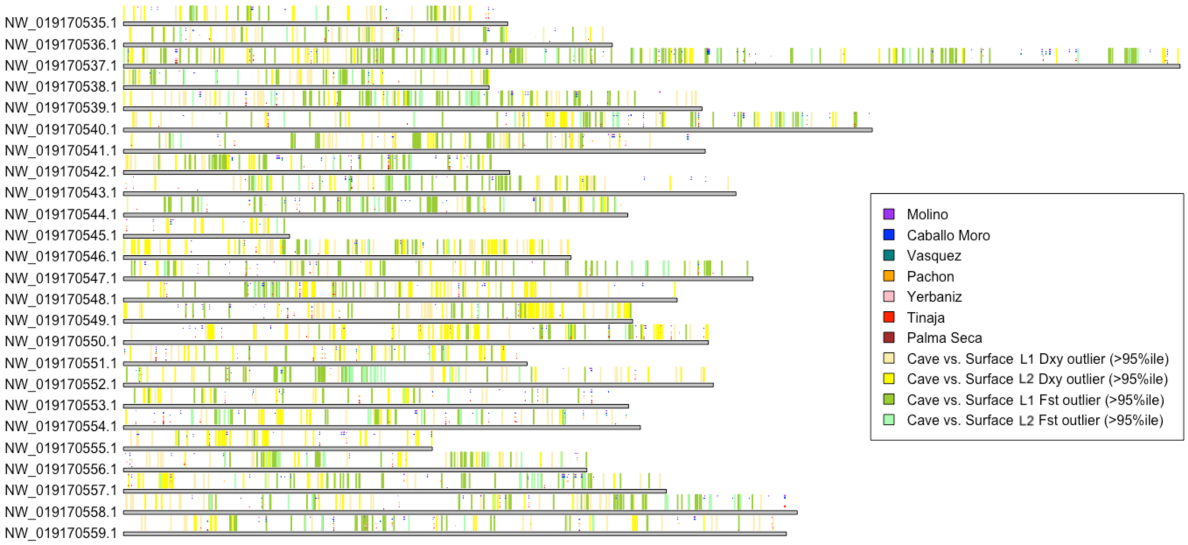

Supplementary Figure 11. Physical locations of putatively adaptive alleles. Shown are locations of adaptive alleles (i.e., genes with selective sweeps and GO terms associated with cave-derived traits) across seven cave populations and outlier windows with exceptionally high divergence (Dxy and Fst values above the 95th percentile, calculated in 50 kb windows) between cave and surface populations (i.e., comparison between all Lineage 1 cave individuals vs. all Lineage 1 surface individuals, and all Lineage 2 cave individuals vs. all Lineage 2 surface individuals).

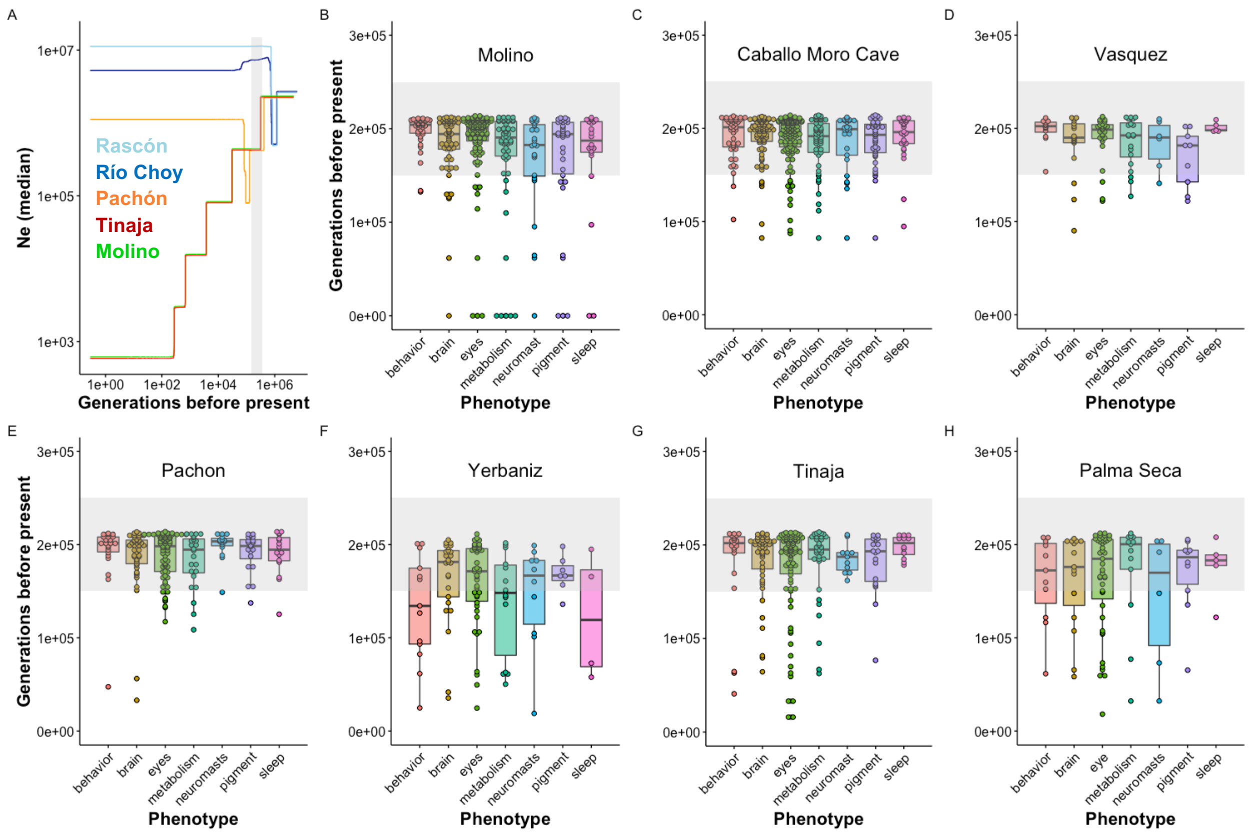

Supplementary Figure 12. Estimated demographic history and ages of cave-derived variants. Estimated demographic history and ages of cave-derived variants within selective sweeps for cave populations for expanded set of phenotypes (including behavior and brain). (A) Stairway plot showing median Ne over time in Pachón (orange), Tinaja (red), and Molino (green) cave populations and Rascón (light blue) and Río Choy (dark blue) surface populations. Bottlenecks for present-day cave populations corresponding to the initial cave invasion by ancestral surface stock are highlighted by a gray rectangle (150,000-250,000 generations before present, spanning the range of previous demographic model-based median estimates for split times between Lineage 1 and Lineage 2 cave and surface lineages from (Herman et al. 2018)). Note a more ancient bottleneck in the two surface populations shown (Rascón and Río Choy) around 800,000 generations before present, likely corresponding to migration into northern Mexico. (B-H) Ages of selective sweeps with GO terms associated with cave-adaptive phenotypes (see Supplementary Table 5) in seven cave populations. Northern lineage caves from the Guatemala regions are shown in B-D. Lineage 2 caves from the El Abra regions are shown in E-H. Gray rectangles span 150,000-250,000 generations before present. For each box plot the horizonal line shows the median variant age, the shaded box spans the 25th to 75th percentile range, and the whiskers span the lowest to highest values that fall within 1.5 * the inter-quartile range. Raw data are shown over the box plots, with each dot representing a single gene. The number of independent biological replicates for each of the seven cave population included in B-H ranged from n = 7 to n = 18 (see Supplementary Table 3). See Supplementary Data 4 for the number of genes in each phenotypic category within each population.

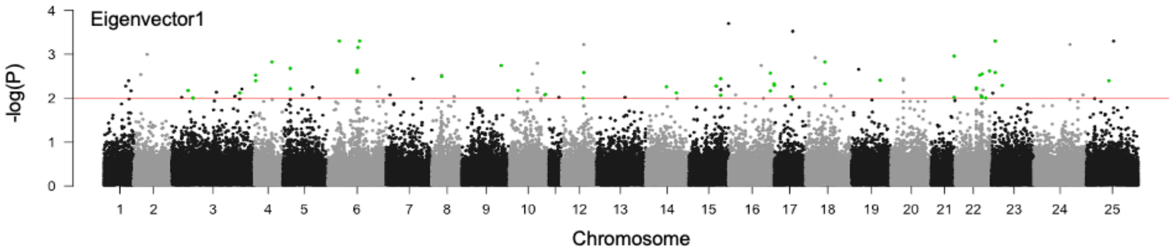

Supplementary Figure 13. Visualization of AFvape-R scan for genomic windows showing a signature of repeated evolution across cavefish lineages. Each point represents a 50 SNP window. Values along the Y axis indicate empirical p-values above the 99th percentile (red line; generated using 10,000 null permutations) for loadings on eigenvector 1 across all 25 chromosomes (see Supplementary Data 6). The 47 windows (overlapping 34 genes) that showed evidence of allele reuse across all seven cave populations are highlighted in green.

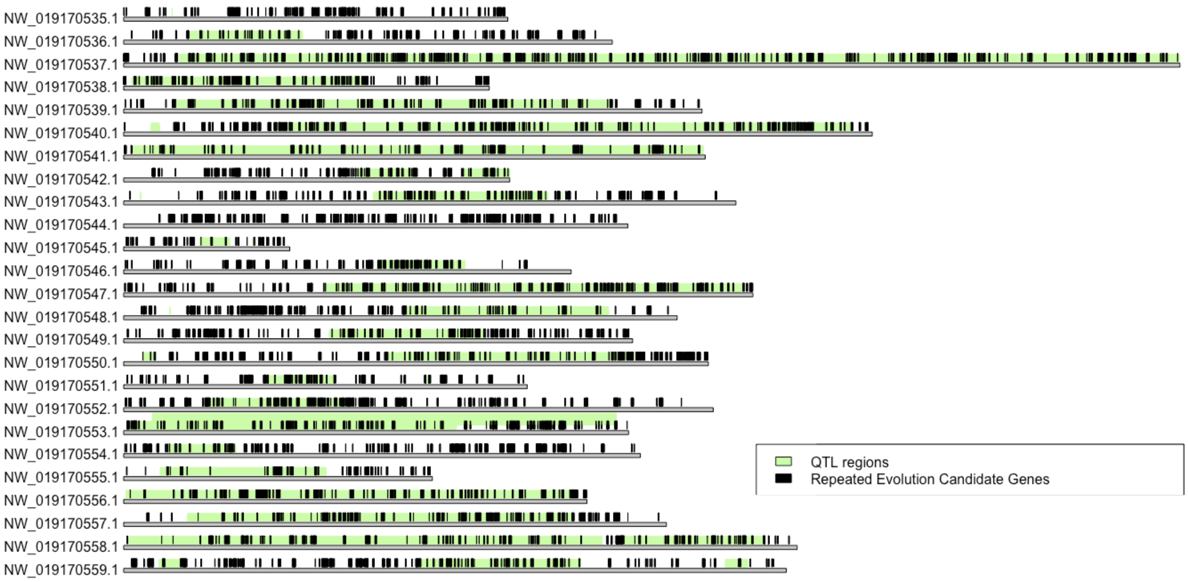

Supplementary Figure 14. Location of QTL regions and repeated evolution candidate genes. Plot of 25 *A. mexicanus* chromosomes with known QTL regions from previous studies highlighted in green and location of 4,085 candidate genes for repeated evolution occurring on an assembled chromosome shown in black. Candidate genes were identified using a scan for parallel selection with AF-vapeR and an overlapping sweeps approach (see main text for details).

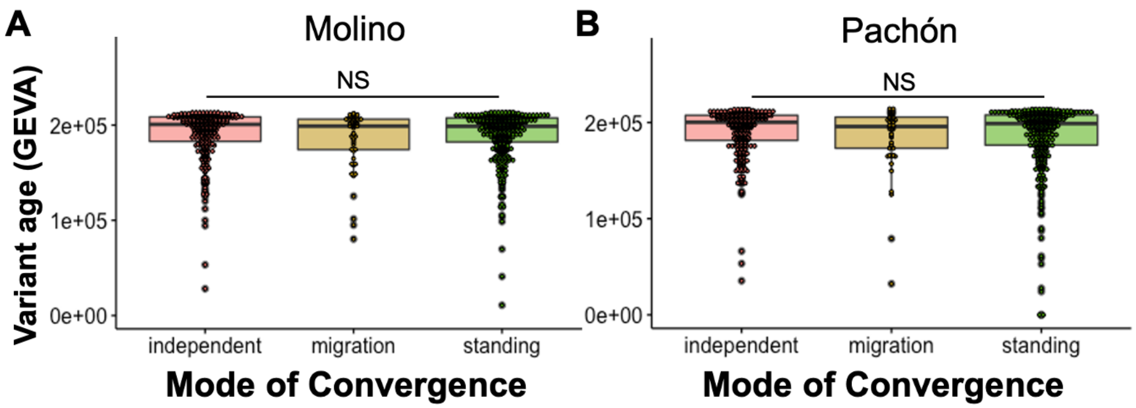

Supplementary Figure 15. Age of derived variants by mode of convergence. Age of derived varinats within selective sweeps (from GEVA) for the 760 overlapping sweep candidate genes do not vary by my mode of repeated evolution (from DMC) in (A) Molino (Lineage 1, left; n = 15) and (B) Pachón (Lineage 2, right; n = 18). For each box plot the horizonal line shows the median variant age, the shaded box spans the 25th to 75th percentile range, and the whiskers span the lowest to highest values that fall within 1.5 * the inter-quartile range. Raw data are shown over the box plots, with each dot representing a single gene. See Supplementary Data 5 for the number of genes in each category within each population. One-way ANOVAs indicated no significant difference (NS) in the estimated timing that derived variants arose among genes falling within different modes of convergence for (A) Molino ($F_{2,717} = 1.46$; $p = 0.23$) and (B) Pachón ($F_{2,700} = 2.31$; $p = 0.10$).

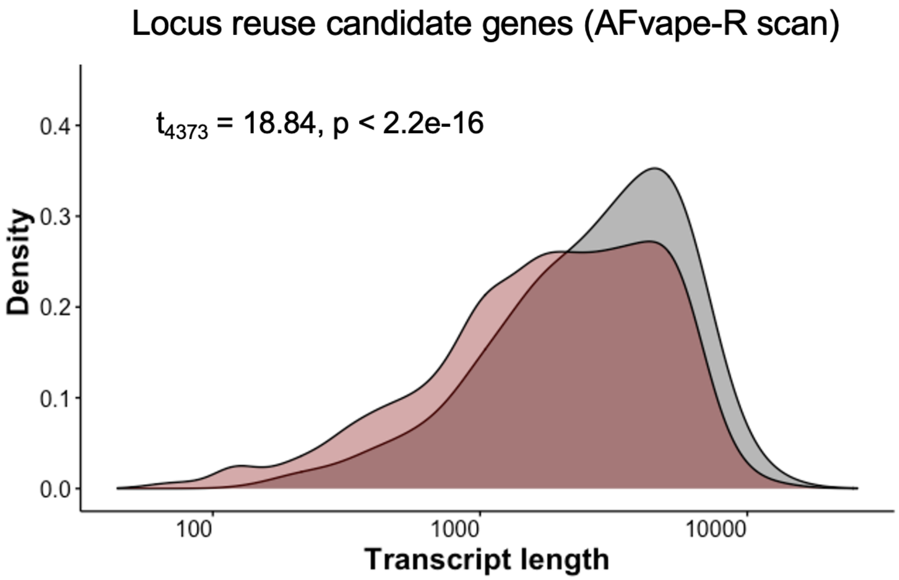

Supplementary Figure 16. Locus reuse candidate gene transcript lengths. Density plot of transcript length for the set candidate genes identified by AF-vapeR as evolving repeatedly via locus reuse across seven cave populations (gray) versus the whole genome (red) (two-sided t-test, t = 18.84, df = 4373.2, $p < 2.2e-16$).

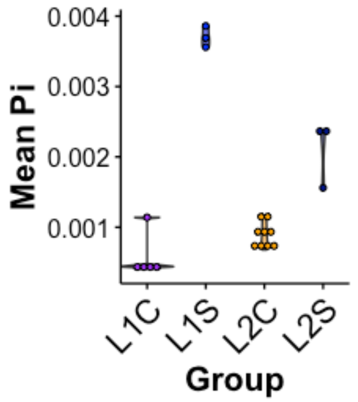

Supplementary Figure 17. Nucleotide diversity within populations. Violin plots depicting the kernel probability density of the mean nucleotide diversity (pi) within each population, categorized by lineage and ecotype. Raw data are also shown, with each dot representing a population mean. Hybrid populations (i.e., Caballo Moro Cave eyed individuals, Arroyo, Chica, Toro, and Subterráneo) and populations with less than 3 samples (i.e., Micos and Jalpan) were excluded. L1C = lineage 1 cave (n = 5), L1S = lineage 1 surface (n = 3), L2C = lineage 2 cave (n = 9), L2S = lineage 2 surface (n = 3).

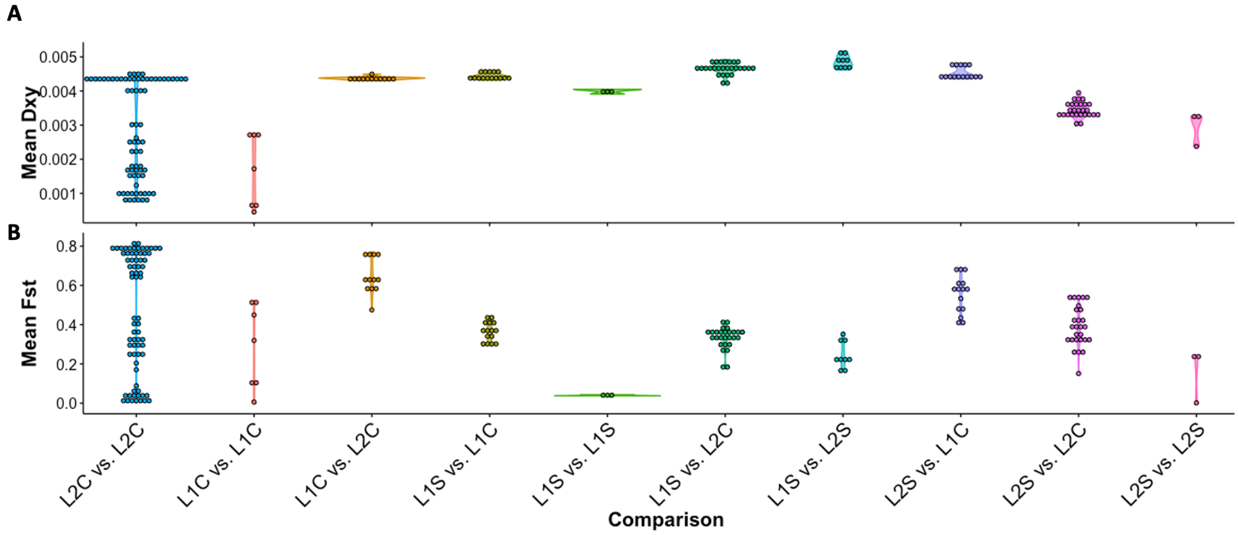

Supplementary Figure 18. Genetic divergence between population pairs. Violin plots depicting the kernel probability density for (A) mean Dxy and (B) mean Fst (calculated in 50 kb windows across the genome) between and within ecotypes and lineages. Raw data is also shown, with each dot representing one pairwise population comparison. Hybrid populations (i.e., Caballo Moro Cave eyed individuals, Arroyo, Chica, Toro, and Subterráneo) and populations with less than 3 samples (i.e., Micos and Jalpan) were excluded. L1C = lineage 1 cave, L1S = lineage 1 surface, L2C = lineage 2 cave, L2S = lineage 2 surface. Sample sizes (number of pairwise comparisons) in each category are as follows; L2C vs. L2C: n = 72; L1C vs. L1C: n = 7; L1C vs. L2C: n = 12; L1S vs. L1C: n = 15; L1S vs. L1S: n = 3; L1S vs. L2C: n = 27; L1S vs. L2S: n = 9; L2S vs. L1C: n = 15; L2S vs. L2C: n = 27; L2S vs. L2S: n = 3.

Supplementary Table 1. Results of D statistic and f4 ratio tests for introgression. *A. nicarageunsis* served as the outgroup. BBAA = derived alleles shared by P1 and P2. ABBA = derived alleles shared by P2 and P3. BABA = derived alleles shared by P1 and P3. Significant p-values (< 0.05) indicate evidence of introgression. An excess of BABA alleles is indicative of introgression between P1 and P2. An excess of ABBA alleles is indicative of introgression between P2 and P3. Subter = Subterranéo. *A. nic* = *A. nicarageunsis.* Key comparisons between Subterranéo and a Lineage 1 cave (i.e., Escondido) and Lineage 2 caves (i.e., Pachón and Tinaja) highlighted.

**P1**	**P2**	**P3**	**P4 (outgroup)**	**Dstat**	**Z-score**	**p-value**	**f4-ratio**	**BBAA**	**ABBA**	**BABA**
Mante	Escondido	Pachón	*A. nic*	0.1027	12.4326	<0.0001	0.0538	10,785.00	7,257.88	5,906.06
Mante	Escondido	Rascón	*A. nic*	0.2058	17.4161	<0.0001	0.1692	13,029.60	6,028.40	3,970.98
Mante	Subter	Escondido	*A. nic*	0.0655	12.2241	<0.0001	0.0308	9,921.08	**8,486.78**	7,443.59
Mante	Escondido	Tinaja	*A. nic*	0.1631	19.5420	<0.0001	0.0781	12,262.50	6,474.62	4,659.12
Escondido	Pachón	Rascón	*A. nic*	0.2047	16.7233	<0.0001	0.2195	7,739.13	6,484.09	4,280.39
Subter	Escondido	Pachón	*A. nic*	0.0367	5.0787	<0.0001	0.0192	11,077.70	6,524.09	6,062.08
Tinaja	Pachón	Escondido	*A. nic*	0.2299	19.2203	<0.0001	0.0569	14,111.20	6,011.75	3,764.41
Subter	Escondido	Rascón	*A. nic*	0.0872	11.3729	<0.0001	0.0771	13,205.80	5,215.82	4,378.82
Rascón	Tinaja	Escondido	*A. nic*	0.1241	20.0780	<0.0001	0.0294	7,072.97	5,427.59	4,229.47
Subter	Escondido	Tinaja	*A. nic*	0.0609	8.9048	<0.0001	0.0297	12,473.50	5,662.26	5,012.16
Rascón	Pachón	Mante	*A. nic*	0.3571	24.2395	<0.0001	0.2522	8,185.19	8,099.02	3,836.95
Mante	Subter	Pachón	*A. nic*	0.0718	11.2772	<0.0001	0.0350	13,325.50	**6,715.01**	5,814.88
Tinaja	Pachón	Mante	*A. nic*	0.2719	20.0060	<0.0001	0.1803	16,274.10	6,511.76	3,727.89
Mante	Subter	Rascón	*A. nic*	0.1294	15.1870	<0.0001	0.0992	15,954.10	5,383.49	4,149.91
Rascón	Tinaja	Mante	*A. nic*	0.1581	16.3223	<0.0001	0.0870	8,933.86	5,400.27	3,925.64
Mante	Subter	Tinaja	*A. nic*	0.1096	15.6274	<0.0001	0.0491	15,046.40	**5,916.42**	4,747.49
Subter	Pachón	Rascón	*A. nic*	0.2700	18.1570	<0.0001	0.2791	8,084.42	7,270.94	4,179.30
Pachón	Tinaja	Rascón	*A. nic*	0.0860	9.7585	<0.0001	0.0800	13,023.70	4,054.58	3,412.69
Tinaja	Pachón	Subter	*A. nic*	0.2400	17.7770	<0.0001	0.0831	15,172.70	6,439.49	3,947.16
Rascón	Tinaja	Subter	*A. nic*	0.1439	17.0269	<0.0001	0.0446	7,923.63	5,592.76	4,185.47

Supplementary Table 2. Number and proportion of 5 kb genomic windows with predictions in each category (neutral, linkedSoft, soft sweep, linkedHard, hard sweep) and total windows with evidence of hard or soft sweeps (all sweeps) from diploS/HIC.

Lineage	Population	Type	n	Total5kbWindowsWithPredictions	neutral	prop neutral	linkedSoft	prop linkedSoft	soft sweeps	prop soft sweeps	linkedHard	prop linkedHard	hard sweeps	prop hard sweeps	all sweeps	prop all sweeps
2	Rascón	Surface	13	215,196	13,979	0.0650	150,102	0.6975	10,449	0.0486	12,074	0.0561	28,592	0.1329	39,041	0.1814
2	Peroles	Surface	9	206,088	58,302	0.2829	95,979	0.4657	38,567	0.1871	5,175	0.0251	8,065	0.0391	46,632	0.2263
1	Choy	Surface	9	212,384	45,929	0.2163	137,976	0.6497	25,319	0.1192	155	0.0007	3,005	0.0141	28,324	0.1334
1	Mante	Surface	10	216,870	14,202	0.0655	181,146	0.8353	9,602	0.0443	1,309	0.0060	10,611	0.0489	20,213	0.0932
1	Caballo Moro	Surface	6	211,924	4,281	0.0202	188,378	0.8889	4556	0.0215	1757	0.0083	12952	0.0611	17,508	0.0826
2	Yerbaniz	Cave	7	99,975	5,995	0.0600	38,735	0.3874	15,874	0.1588	32,876	0.3288	6,495	0.0650	22,369	0.2237
2	Tinaja	Cave	17	208,538	12,165	0.0583	45,369	0.2176	18,459	0.0885	103,452	0.4961	29,093	0.1395	47,552	0.2280
2	Palma Seca	Cave	8	76,410	4279	0.0560	26869	0.3516	11872	0.1554	28072	0.3674	5318	0.0696	17,190	0.2250
2	Pachón	Cave	18	200,799	7,727	0.0385	19,919	0.0992	9,217	0.0459	132,103	0.6579	31,833	0.1585	41,050	0.2044
1	Vasquez	Cave	8	53,484	2,786	0.0521	10,511	0.1965	5,061	0.0946	29,352	0.5488	5,774	0.1080	10,835	0.2026
1	Caballo Moro	Cave	12	189,653	7079	0.0373	35926	0.1894	15213	0.0802	104216	0.5495	27219	0.1435	42,432	0.2237
1	Molino	Cave	15	190,397	2,030	0.0107	8,676	0.0456	3,524	0.0185	144,262	0.7577	31,905	0.1676	35,429	0.1861

Supplementary Table 3. Breakdown of number of 5 kb windows (out of 267,048 total in the assembly) and genes (out of 26,698 total) included in diploS/HIC analyses for each population. Some 5 kb windows were skipped by diploS/HIC during the prediction step due to missing data or lack of SNPs.

Lineage	Population	Type	n	Total 5kb Windows with Predictions	Prop 5kb Windows with Predictions	Genes with Predictions	Prop Genes with Predictions
1	Choy	Surface	9	212,384	0.795	22,866	0.856
1	Mante	Surface	10	216,870	0.812	23,222	0.870
1	Caballo Moro	Surface	6	211,924	0.794	23,205	0.869
2	Peroles	Surface	9	206,088	0.772	22,390	0.839
2	Rascón	Surface	13	215,196	0.806	23,117	0.866
1	Vasquez	Cave	8	53,484	0.200	7,741	0.290
1	Caballo Moro	Cave	12	189,653	0.710	21,045	0.788
1	Molino	Cave	15	190,397	0.713	21,251	0.796
2	Yerbaniz	Cave	7	99,975	0.374	13,130	0.492
2	Tinaja	Cave	17	208,538	0.781	22,737	0.852
2	Palma Seca	Cave	8	76,410	0.286	11,034	0.413
2	Pachón	Cave	18	200,799	0.752	22,136	0.829

Supplementary Table 4. Number of candidate genes for adaptive evolution across seven cave populations (i.e., evidence of a soft or hard selective sweep identified by diploS/HIC in the cave population but no sweep in a same-lineage surface population) and number of those candidate genes with a GO term associated with cave-derived phenotypes.

Cave population	Lineage	Sweep in cave, neutral in same-lineage surface	Sweep in cave, neutral in same-lineage surface, and relevant GO term
Molino	1	5494	285
Vasquez	1	1777	82
Caballo Moro	1	6238	315
Pachón	2	3903	206
Yerbaniz	2	2328	100
Tinaja	2	4389	217
Palma Seca	2	1984	103

Supplementary Table 5. Keywords related to known cave-derived phenotypes identified in the GO terms associated with each candidate gene. Candidate genes were defined as a gene with a soft or hard sweep in a given cave population but no sweep in the same-lineage surface population, and a fixed or nearly fixed variant in the cave population. R = regressive traits. C = constructive traits.

**Phenotypic category**	**Keywords (searched for in GO terms)**
Eye development (R)	eye, lens, optic, iris, retina, detection of light stimulus, visual, photoreceptor
Pigment (R)	pigment, melanin
Sleep (R)	sleep, circadian, rhythm, photoperiod, response to light stimulus
Metabolism (C)	insulin, glucose, body fat, fat pad, adipose
Neuromasts (C)	neuromast, hair cell, lateral line
Behavior	behavior
Brain	brain, hypothalamus, amygdala, telencephalon

Supplementary Table 6. Results of Kruskal-Wallis rank sum tests on estimated timing of selective sweeps (from GEVA) in genes with GO terms associated with cave-derived phenotypic categories (i.e., eyes, pigment, sleep, metabolism, brain, behavior, neuromasts; see Supplementary Table 5, Supplementary Figure 11) within each of the seven cave populations examined.

Cave Population	Chi-square	df	P
Molino	10.18	6	0.12
Caballo Moro	3.18	6	0.79
Vasquez	8.47	6	0.21
Pachón	3.96	6	0.68
Yerbaniz	4.74	6	0.58
Tinaja	3.54	6	0.74
Palma Seca	2.27	6	0.89

Supplementary Table 7. Results of Wilcoxon rank sum tests on estimated timing of selective sweeps (from GEVA) in genes with GO terms associated with regressive traits (i.e., eyes, pigment, sleep) versus constructive traits (i.e., metabolism, neuromasts; see Supplementary Table 5, Fig. 2b-h) within each of the seven cave populations examined.

**Cave Population**	**W**	**P**
Molino	4996	0.94
Caballo Moro	6423	0.77
Vasquez	530	0.52
Pachón	2060	0.44
Yerbaniz	530	0.90
Tinaja	2487	0.50
Palma Seca	577	0.39

Supplementary Table 8. Effect sizes (Cohen's *d*) for comparison of transcript length in candidate genes for repeated evolution in caves, broken down by mode of repeated evolution predicted with DMC, compared to the whole genome.

**Predicted Mode of Repeated Evolution**	**Cohen's *d***
Independent	0.524
Migration	0.264
Standing	0.256

Supplementary Table 9. GATK filters applied to variant and invariant sites. QD = QualByDepth. FS = FisherStrand. MQ = RMSMappingQuality.

**Invariant sites**	**SNPs**	**Mixed/indels**
QD < 2.0	QD < 2.0	QD < 2.0
FS > 60.0	FS > 200.0	FS > 200.0
MQ < 40.0	ReadPosRankSum < -20.0	ReadPosRankSum < -20.0

Supplementary Table 10. Parameter values specified to DMC. sels = selection coefficient; times = standing time; gs = frequency of the standing variant; migs = migration rate; proportion of migrants between populations each generation

DMC Parameter	Values Specified
sels	0.0001, 0.0010, 0.0100, 0.0200, 0.0300, 0.0400, 0.0500, 0.0600, 0.0700, 0.0800, 0.0900 0.1000, 0.1100, 0.1200, 0.1300, 0.1400, 0.1500, 0.2000, 0.2500, 0.3000, 0.4000, 0.5000, 0.6000
times	5, 50, 100, 500, 1000, 10000, 1000000
gs	0.00005, 0.0001, 0.001, 0.01, 0.1
migs	0.00001, 0.001, 0.1, 0.5, 1.0

Supplementary References

Charlesworth, B. 1998. Measures of divergence between populations and the effect of forces that reduce variability. Molecular biology and evolution **15**:538-543.

Elliott, W. R. 2018. The *Astyanax* caves of Mexico: Cavefishes of Tamaulipas, San Luis Potosí, and Guerrero. Association for Mexican Cave Studies.

Gross, J. B., R. Borowsky, and C. J. Tabin. 2009. A novel role for *Mc1r* in the parallel evolution of depigmentation in independent populations of the cavefish *Astyanax mexicanus*. PLoS genetics **5**:e1000326-e1000326.

Gutenkunst, R., R. Hernandez, S. Williamson, and C. Bustamante. 2010. Diffusion approximations for demographic inference: DaDi. Nature precedings:1-1.

Herman, A., Y. Brandvain, J. Weagley, W. R. Jeffery, A. C. Keene, T. J. Y. Kono, H. Bilandžija, R. Borowsky, L. Espinasa, K. O'Quin, C. Ornelas-García, M. Yoshizawa, B. Carlson, E. Maldonado, J. B. Gross, R. A. Cartwright, N. Rohner, W. Warren, and S. E. McGaugh. 2018. The role of gene flow in rapid and repeated evolution of cave related traits in Mexican tetra, *Astyanax mexicanus*. Molecular ecology **27**:4397– 4416

Liu, X., and Y.-X. Fu. 2020. Stairway Plot 2: demographic history inference with folded SNP frequency spectra. Genome biology **21**:1-9.

Malinsky, M., H. Svardal, A. M. Tyers, E. A. Miska, M. J. Genner, G. F. Turner, and R. Durbin. 2018. Whole-genome sequences of Malawi cichlids reveal multiple radiations interconnected by gene flow. Nature ecology & evolution **2**:1940-1955.

Mitchell, R. W., W. H. Russell, and W. R. Elliott. 1977. Mexican eyeless characin fishes, genus *Astyanax:* Environment, distribution, and evolution. Texas Tech Press, Texas.

Moran, R. L., J. B. Jaggard, E. Y. Roback, K. Alexander, R. Nicolas, J. E. Kowalko, C. P. Ornelas-García, S. E. McGaugh, and A. C. Keene. 2022. Hybridization underlies localized trait evolution in cavefish. iScience:103778.

Panaram, K., and R. Borowsky. 2005. Gene flow and genetic variability in cave and surface populations of the Mexican tetra, *Astyanax mexicanus* (Teleostei: Characidae). Copeia **2005**:409-416.
