# Supplementary material for: Selection-driven trait loss in independently evolved cavefish populations
Source: Nat Commun. 2023 May 3;14:2557. doi: 10.1038/s41467-023-37909-8 (PMC10156726; doi:10.1038/s41467-023-37909-8)
Supplement: Supplementary file 3 — Reporting Summary [file 41467_2023_37909_MOESM3_ESM.pdf]

nature portfolio

Corresponding author(s): Rachel L. Moran

Last updated by author(s): Mar 16, 2023

Reporting Summary

Nature Portfolio wishes to improve the reproducibility of the work that we publish. This form provides structure for consistency and transparency in reporting. For further information on Nature Portfolio policies, see our Editorial Policies and the Editorial Policy Checklist.

Statistics

For all statistical analyses, confirm that the following items are present in the figure legend, table legend, main text, or Methods section.

n/a	Confirmed	
☐	☒	The exact sample size (*n*) for each experimental group/condition, given as a discrete number and unit of measurement
☐	☒	A statement on whether measurements were taken from distinct samples or whether the same sample was measured repeatedly
☐	☒	The statistical test(s) used AND whether they are one- or two-sided<br>*Only common tests should be described solely by name; describe more complex techniques in the Methods section.*
☒	☐	A description of all covariates tested
☐	☒	A description of any assumptions or corrections, such as tests of normality and adjustment for multiple comparisons
☐	☒	A full description of the statistical parameters including central tendency (e.g. means) or other basic estimates (e.g. regression coefficient) AND variation (e.g. standard deviation) or associated estimates of uncertainty (e.g. confidence intervals)
☐	☒	For null hypothesis testing, the test statistic (e.g. *F*, *t*, *r*) with confidence intervals, effect sizes, degrees of freedom and *P* value noted<br>*Give P values as exact values whenever suitable.*
☒	☐	For Bayesian analysis, information on the choice of priors and Markov chain Monte Carlo settings
☐	☒	For hierarchical and complex designs, identification of the appropriate level for tests and full reporting of outcomes
☐	☒	Estimates of effect sizes (e.g. Cohen's *d*, Pearson's *r*), indicating how they were calculated

Our web collection on statistics for biologists contains articles on many of the points above.

Software and code

Policy information about availability of computer code

Data collection	Ensembl Biomart v104
Data analysis	Custom scripts were used for calculating population genomic statistics (Pi, Dxy, Fst) at each basepair in the genome and summary statistics for each gene and are available on GitHub under DOI: 10.5281/zenodo.7706730118 and DOI: 10.5281/zenodo.7706736119.<br>Additional software used:<br>Cutadapt v1.2.1<br>Trimmomatic v0.30<br>bwa v0.7.4<br>Picard v2.3.0<br>samtools v1.7<br>Genome Analysis Tool Kit (GATK) v3.7.0, v3.8.0, and v4.1.4<br>vcftools v0.1.15<br>ADMIXTURE v1.3.0<br>Plink v1.90<br>Treemix v1.13<br>treemixVarianceExplained.R (https://github.com/darencard/RADpipe/blob/master/treemixVarianceExplained.R)<br>Dsuite v0.4<br>Loter v0.1<br>get_fixed_site_gts.rb (https://github.com/mmatschiner/tutorials/blob/master/analysis_of_introgression_with_snp_data/src/get_fixed_site_gts.rb)

March 2021

plot_fixed_site_gts.rb (https://github.com/mmatschiner/tutorials/blob/master/analysis_of_introgression_with_snp_data/src/plot_fixed_site_gts.rb)
popgenWindows.py (https://github.com/simonhmartin/genomics_general/blob/master/popgenWindows.py)
R v3.6.3 & v4.0.2
ShinyGO v74 (http://bioinformatics.sdstate.edu/go74/
diploS/HIC v0.333 (https://doi.org/10.1534/g3.118.200262)
discoal v0.1.5 (https://doi.org/10.1093/bioinformatics/btw556)
AUGUSTUS (v3.3.3)
ASTRAL-MP (v5.15.4)
BUSCO (v2.0)
IQ-TREE (v1.6.12)
PAUP (v4.0a)

For manuscripts utilizing custom algorithms or software that are central to the research but not yet described in published literature, software must be made available to editors and reviewers. We strongly encourage code deposition in a community repository (e.g. GitHub). See the Nature Portfolio guidelines for submitting code & software for further information.

Data

Policy information about availability of data

All manuscripts must include a data availability statement. This statement should provide the following information, where applicable:

- Accession codes, unique identifiers, or web links for publicly available datasets
- A description of any restrictions on data availability
- For clinical datasets or third party data, please ensure that the statement adheres to our policy

All sequencing data generated for this project is available under PRJNA558458. Accession numbers for each sample used in sequence analyses are provided in Supplementary Data 1. The surface fish Astyanax mexicanus genome assembly, Astyanax_mexicanus-2.0 (accession # GCF_000372685.2) is available on NCBI. Ensembl's Biomart (v104) is available at https://www.ensembl.org/biomart/martview/. Dataset containing population genetic summary statistics for each gene in the genome is available at Dryad (https://doi.org/10.5061/dryad.3xsj3txmf).

Human research participants

Policy information about studies involving human research participants and Sex and Gender in Research.

Reporting on sex and gender	NA
Population characteristics	NA
Recruitment	NA
Ethics oversight	NA

Note that full information on the approval of the study protocol must also be provided in the manuscript.

Field-specific reporting

Please select the one below that is the best fit for your research. If you are not sure, read the appropriate sections before making your selection.

☐ Life sciences ☐ Behavioural & social sciences ☒ Ecological, evolutionary & environmental sciences

For a reference copy of the document with all sections, see nature.com/documents/nr-reporting-summary-flat.pdf

Ecological, evolutionary & environmental sciences study design

All studies must disclose on these points even when the disclosure is negative.

Study description	The study does not involve experiments that generated quantitative data. We conducted whole genome sequencing and population genomic analyses to investigate the repeated evolution of cave adaptation in Mexican tetras (Astyanax mexicanus).
Research sample	We sampled cave and surface ecotypes of the Mexican tetra, Astyanax mexicanus, across 18 cave and eight surface populations throughout their range in northeastern and central Mexico (Figure 1B). The Mexican tetra was chosen becasue it is a model system for studying repeated evolution. Specific populations included are shown in Fig. 1b. We made an effort to include cave populations that spanned each of the three cave systems where this species is found (Micos Caves, El Abra Caves, and Guatemala Caves) and samples surface populations from multiple drainages throughout the range of the species.
Sampling strategy	We aimed to sequence at least 10 individuals per population for those used in selection analyses. We were limited by the amount of samples we were able to collect in the field and how many individuals total we had the budget to sequence. We aimed to balance

	number of populations included in the analysis with number of samples per population. Our sample sizes were based on previous work in this system (Herman et al. 2018 Molecular Ecology, Moran et al. 2021 iScience).
Data collection	Data resulting from computational analyses were recorded by R. Moran in Microsoft Excel sheets. Data were deposited in shared lab drives and made available as supplementary materials.
Timing and spatial scale	Fish were collected from 2015-2019. Collection took place during the dry season, except for 2016, when fish were collected from Pachón cave during the rainy season. We made an effort to collect from populations within each of the three cave regions in Mexico where A. mexicanus occurs. The dates of collection, associated permits, and population are as follows:<br><br>SEMARNAT SGPA/DGVS/2438/15<br>The collection dates were: February 14th, 2015, Chica<br>February 15th, 2015, Pachón<br>February 16th, 2015, Sabinos<br>April 28t, 2015, Micos<br><br>SEMARNAT SGPA/DGVS/2438/16<br>The collection dates were: February 21st- March 3rd, 2016, August 5th and 8th, Pachón, Jineo, Escondido, Molino, and Jos.<br><br>SEMARNAT SGPA/DGVS/05389/17<br>The collection dates were: January 30th- May 19th, 2017, Palma Seca, Vazquez, Montecillos, Japones, Yerbaniz, Caballo Moro, and Tigre<br><br>SEMARNAT SGPA/DGVS/05389/18<br>The collection dates were: February, 16-20st, 2018, Jalpan<br><br>SEMARNAT SGPA/DGVS/1893/19<br>The collection dates were: February 19th, 2019, Arroyo<br>February 19th, 2019, Micos
Data exclusions	Sample Rascón _6 was excluded from test for introgression in Subterráneo because ADMIXTURE indicated that it was likely an early generation hybrid. Including early generation hybrids would affect inferences of selection and population genetic statistics. Early generation hybrids are typically excluded from such analyses for these reasons (e.g., see Herman et al. 2018 Molecular Ecology).
Reproducibility	Experiments were not conducted. Raw genomic data and code used in all analyses are provided to enable reproducibility.
Randomization	This is not relevant to our study. Experiments were not conducted. Population-level population genomic analyses were conducted, and individuals collected together at a single location were grouped together for analyses and considered as members of the same population.
Blinding	Blinding was not relevant during data acquisition and analysis of population genetic data because individuals within each population needed to be grouped together and we needed to consider ecotype and lineage in the analyses. We used unbiased whole-genome approaches to infer phylogenetic relationships among populations and to identify regions of the genome under selection.

Did the study involve field work? ☐ Yes ☒ No

Reporting for specific materials, systems and methods

We require information from authors about some types of materials, experimental systems and methods used in many studies. Here, indicate whether each material, system or method listed is relevant to your study. If you are not sure if a list item applies to your research, read the appropriate section before selecting a response.

Materials & experimental systems

n/a	Involved in the study
☒	☐ Antibodies
☒	☐ Eukaryotic cell lines
☒	☐ Palaeontology and archaeology
☐	☒ Animals and other organisms
☒	☐ Clinical data
☒	☐ Dual use research of concern

Methods

n/a	Involved in the study
☒	☐ ChIP-seq
☒	☐ Flow cytometry
☒	☐ MRI-based neuroimaging

Animals and other research organisms

Policy information about studies involving animals; ARRIVE guidelines recommended for reporting animal research, and Sex and Gender in Research

Laboratory animals	Study did not involve laboratory animals.
Wild animals	Adult (>6 months of age) Astyanax mexicanus were collected with hand held dip nets during the dry season during the years 2015-2019, and were transported back to the IBUNAM fish facility in aerated coolers. Fin clips were taken in July and August of 2019 from the fish maintained at the IBUNAM. For the collection of cave specimens, permission was obtained from the competent authorities (SEMARNAT SGPA/DGVS/2438/15-16, SGPA/DGVS/05389/17, and SGPA/DGVS/1893/19). Thirty-nine individuals were euthanized in cold water then cataloged and deposited in the National Fish Collection of the Institute of Biology, U.N.A.M. (LANABIO, IB-UNAM, Mexico City) under catalog number CNPE23853- 23892. The remaining fish were maintained in the lab at IBUNAM for use in other studies.
Reporting on sex	Sex based analyses were not included. Sex is often not reported as a covariate in Astyanax literature (see Protas et al., 2006, 2008; Yoshizawa et al., 2010, 2015; Moran, Softley and Warrant, 2014; Carlson and Gross, 2018; Frøland Steindal et al., 2018). Where sex has been tested as a factor in the Astyanax literature, there is often little difference between males and females in phenotypes or behavior. Females are larger than males (Protas et al., 2008), but none of the traits associated with QTL examined in Protas et al. (2008) were noted to be impacted by sex (eye size, melanophore number, relative condition, weight loss, tooth count, peduncle depth, fin placement, anal fin rays, SO3 width, number of thoracic ribs, and chemical sense).
Field-collected samples	Fish were collected using dip nets and fin clips were taken on site for use in DNA extraction. Fish were either immediately released back at their collection site.
Ethics oversight	The ethical treatment of animals collected for this study was in compliance with the National Aquaculture and Fisheries Commission of Mexico permit PPF/DGOPA - 106 / 2013 and the Secretariat of Environment and Natural Resources permits SGPA/DGVS/2438/15, SGPA/DGVS/2438/16, SGPA/DGVS/05389/17, SGPA/DGVS/05389/18, and SGPA/DGVS/1893/19 to P. Ornelas-García.

Note that full information on the approval of the study protocol must also be provided in the manuscript.
