# Supplementary material for: Selection-driven trait loss in independently evolved cavefish populations
Source: Nat Commun. 2023 May 3;14:2557. doi: 10.1038/s41467-023-37909-8 (PMC10156726; doi:10.1038/s41467-023-37909-8)
Supplement: Supplementary file 4 — Description of Additional Supplementary Files [file 41467_2023_37909_MOESM4_ESM.docx]

**Description of Additional Supplementary Files**

Supplementary Data 1.

Description: Sample IDs, sequencing information, coverage, and raw and clean read counts.

Supplementary Data 2.

Description: Lists of genes with a sweep in a cave population and neutral evolution in a same-lineage surface population and results of accompanying GO enrichment analyses (using the Gene Ontology Consortium online tool, http://geneontology.org) for individual populations and genes with overlapping sweeps in at least one population from both cavefish lineages.

Supplementary Data 3.

Description: GO enrichment and DMC results. List of genes with sweeps in two surface populations (Lineage 1: Mante , Lineage 2: Rascón) and neutral evolution in three cave populations (Lineage 1: Molino, Lineage 2: Pachón and Tinaja), results of GO enrichment analysis (using the Gene Ontology Consortium online tool, http://geneontology.org) for individual populations, and results of DMC (Distinguishing Modes of Convergence) analysis.

Supplementary Data 4.

Description: GEVA sweep ages for putatively adaptive alleles. Genes included had evidence of a selective sweep in a cave population and neutral evolution in a corresponding same-lineage surface population, and a GO term annotation associated with a cave-derived trait category; see Supplementary Table 5) in each of the seven cave populations analyzed.

Supplementary Data 5.

Description: DMC results for the candidate genes for repeated evolution in caves. This includes 794 genes total, 760 from overlapping sweeps approach, 150 of which were classified as multiparallel (locus reuse) by AF-vapeR; 34 classified as full parallel (allele reuse) by AF-vapeR and for “control” set of 172 genes with evidence of overlapping sweeps in surface populations (Lineage 1: Mante; Lineage 2: Rascón) and neutral evolution in Pachón, Tinaja, and Molino caves. Dxy-based allele split times (between cave and surface alleles) are provided for all 794 candidate genes for repeated evolution in caves. GEVA-based sweep ages are provided for the 760 overlapping sweep candidate genes for repeated evolution in caves.

Supplementary Data 6.

Description: AF-vapeR results. The first tab includes all significant windows (above the 99th percentile, P < 0.01). Full parallel windows had significant loadings on Eigenvector 1. Multiparallel windows had significant loadings on Eigenvectors 1 and 2. Divergent (nonparallel) windows had significant loadings on Eigenvectors 1, 2, and 3. Windows with significant antiparallel loadings on any eigenvector were discarded from further analysis. The second and third tabs list genes within significant windows of interest (full parallel and multiparallel, respectively).

Supplementary Data 7.

Description: ShinyGO (http://bioinformatics.sdstate.edu/go74/) gene ontology enrichment analysis results for candidate genes for repeated evolution in caves (AF-vapeR allele reuse and locus reuse and overlapping sweep candidate genes) and for genes showing a pattern of repeated evolution in surface populations.

Supplementary Data 8.

Description: Information on the number of invariant sites and variant sites (including SNPs, indels, and mixed sites) per chromosome.

Supplementary Data 9.

Description: Mean, median, min, and max Pi within each population and Fst and Dxy between each pair of populations calculated in 50 kb w
